# Factors Influencing the Abundance of the Side Population in a Human Myeloma Cell Line

**DOI:** 10.1155/2011/524845

**Published:** 2011-09-22

**Authors:** Sui-Lin Mo, Jia Li, Yen S. Loh, Ross D. Brown, Adrian L. Smith, Yuling Chen, Douglas Joshua, Basil D. Roufogalis, George Q. Li, Kei Fan, Michelle C. H. Ng, Daniel Man-yuen Sze

**Affiliations:** ^1^The First Affiliated Hospital, Sun Yat-sen University, Guangzhou 510080, China; ^2^Faculty of Pharmacy, The University of Sydney, Sydney, NSW 2006, Australia; ^3^Institute of Haematology, Royal Prince Alfred Hospital, Sydney, NSW 2050, Australia; ^4^Centenary Institute of Cancer Medicine and Cell Biology, The University of Sydney, Sydney, NSW 2006, Australia; ^5^Faculty of Medicine, The University of New South Wales, Sydney, NSW 2052, Australia; ^6^Department of Health Technology and Informatics, The Hong Kong Polytechnic University, Hung Hom, Kowloon, Hong Kong

## Abstract

Side population (SP) refers to a group of cells, which is capable to efflux Hoechst 33342, a DNA-binding dye. SP cells exist both in normal and tumor tissues. Although SP abundance has been used as an indicator for disease prognostic and drug screening in many research projects, few studies have systematically examined the factors influencing SP analysis. In this study we aim to develop a more thorough understanding of the multiple factors involved in SP analysis including Hoechst 33342 staining and cell culture. RPMI-8226, a high SP percentage (SP%) human myeloma cell line was employed here. The results showed that SP% was subject to staining conditions including: viable cell proportion, dye concentration, staining cell density, incubation duration, staining volume, and mix interval. In addition, SP% was highest in day one after passage, while dropped steadily over time. This study shows that both staining conditions and culture duration can significantly affect SP%. In this case, any conclusions based on SP% should be interpreted cautiously. The relation between culture duration and SP% suggests that the incidence of SP cells may be related to cell proliferation and cell cycle phase. Maintaining these technical variables consistently is essential in SP research.

## 1. Introduction

Side population (SP) cells were first described as a subset of adult mouse bone marrow with enriched hematopoietic stem cells (HSCs) [1, 2]. This subset was characterized by its ability to rapidly efflux the Hoechst 33342 DNA-binding dye and therefore shows a Hoechst 33342^lo^ profile on flow cytometry. Specifically they display a distinct staining pattern, based on the phenomenon of a differential emission of blue (450 nm) versus red (670 nm) emission fluorescence upon UV excitation, such that SP appears as a tiny population on the lower left-hand side of a red (*x*)-blue (*y*) flow cytometry scattergram. This differential blue-red emission allows clear identification of a cell population that locates sideways from the diagonal and was thus named “side” population. Recent studies have shown the presence of SP cells in many types of cancer including ovarian cancer, glioblastoma cancer, lung cancer, nasopharyngeal cancer, gastrointestinal cancers hepatocellular carcinoma, mesenchymal tumors, and multiple myeloma [3–11]. SP cells in these types of cancer showed significantly higher potential to initiate tumor in NOD/SCID mice than their non-SP counterparts. They are also more likely to be resistant to certain anticancer drugs than non-SP cells. These results raised the significance of SP, which would reveal a new insight for cancer research. 

Although studies indicated that SP% varied among different types of cancer and from sample to sample as well [3–11], some studies have used a quantitation of SP% as an indicator for purposes such as prognostics and efficacy of anti cancer drugs [3, 8, 9, 11]. It is therefore important to understand the factors that affect SP%; otherwise, uncontrolled experimental conditions would result in nonreproducible and inconsistent results. To date, some studies have indicated that factors such as flow cytometry setting and gating strategy, staining procedures, and cell viability issues affect the SP% significantly [1, 12–15]; however, few investigations have approached this issue in a systemic way. 

In this study, using the human myeloma cell line RPMI-8226 as a convenient cellular model system *in vitro*, we systematically explored the variables involved with optimization and standardization of Hoechst 33342 staining variables such as dye concentration, cell density, staining duration, staining volume, and mix interval during staining as well as cell viability prior to flow cytometry (FCM) analysis. Importantly, we found that the time after cell subculture is the single most important factor affecting the SP% and this has not been reported before. In summary, this study suggests that both the Hoechst staining and subculture duration affect the proportion of SP. Hence, the conclusions of any other studies based on the change of SP% should be interpreted cautiously. Attempts to maintain these factors in a more consistent manner is instrumental for building reliable platforms for drug screening which target SP. 

## 2. Materials and Methods

### 2.1. Cell Culture

The human myeloma cell line RPMI-8226 was obtained from the American Type Culture Collection (Rockville, MD). RPMI-8226 cells were maintained in RPMI-1640 (Invitrogen, Carlsbad, CA) containing 100 u/mL of penicillin (Invitrogen), 100 *μ*g/mL of streptomycin (Invitrogen), and 10% fetal bovine serum (FBS) (Sigma-Aldrich, St Louis, MO). Cells were cultured in T75 or T25 flasks kept in a humidified incubator with 5% CO_2_ at 37°C. The cells were seeded at the density of 0.2 × 10^6^ cells/mL.

### 2.2. Cell Staining

RPMI-8226 cells were harvested after culture for different periods of time and then stained with Hoechst 33342 dye (Invitrogen). Briefly, after discarding culture medium, cells were suspended in the staining medium RPMI1640^+^ containing 2% FBS and 10 mM HEPES buffer (Invitrogen). Live cell number was counted at least twice and adjusted to a final cell density of 1 × 10^6^ cells/mL by adding appropriate volume of warm staining medium. Hoechst 33342 water solution (1 mg/mL) was then added to make a final concentration of 10 *μ*g/mL followed by incubation in a water bath at 37°C for 90 min with shaking every 30 min (except in the experiments that specifically address the shaking factor). To help gate SP on flow cytometry, samples treated with 1 *μ*g/mL fumitremorgin C (FTC) (Sigma, CITY), an ABCG2 transporter inhibitor, were included during the entire staining procedure as controls. Once incubation finished, samples were immediately put on ice to stop dye efflux. Subsequently, the cells were centrifuged for 5 min at 300 g at 4°C and resuspended in 100 *μ*L of ice-cold Hanks' Balanced Saline Solution (HBSS) (Invitrogen) containing 2% FBS, 100 u/mL of penicillin, 100 *μ*g/mL of streptomycin, and 10 mM HEPES. The samples were kept on ice before flow cytometry analysis. Propidium iodide (PI) (Sigma) solution was added at a final concentration of 2 *μ*g/mL to exclude dead cells just before flow analysis.

### 2.3. Exploration of Factors Affecting SP%

To explore how cell culture affected SP%, RPMI-8226 cells were maintained in culture medium for a range of time prior to being harvested for downstream staining. Preparation of the samples in order to examine the effect of culture time course on SP% was as follows: RPMI-8226 cells were seeded to four T-25 flasks at 0.2 × 10^6^ cells/mL under the same conditions. The cells were cultured for up to four days and then harvested at the end of 1, 2, 3, and 4 days, respectively. Cells were counted using trypan blue at least twice to adjust same staining density (1 × 10^6^ cells/mL) across different experiment days. Two batches of samples were prepared from different but close passages of cells. Cells were subsequently stained under the conditions described as below. A subsequent independent experiment was further performed at the laboratory of Hong Kong Polytechnic University with different batches of RPMI-8226 passage and Hoechst dye. 

We also investigated the SP% associated with an array of different staining conditions: Hoechst 33342 concentration (ranging from 1–50 *μ*g/mL), cell density (0.25 × 10^6^–2.00 × 10^6^ cells/mL), volume (100–2000 *μ*L), duration (30–180 min), shaking interval (10–90 min), and viable cell proportion (20–80%). Only one factor was varied and examined each time, while the others were kept constant. The standard protocol described before was employed for the staining procedure except for the testing of the effects of cell viability prior to FCM analysis on SP%. For those experiments, two tubes of cell suspensions with a density of 2 × 10^6^ cells/mL were prepared in staining medium, one of which was treated by incubation at 60°C for 20 min until all the cells died. We then mixed 0.1, 0.2, 0.3, 0.4, 0.5 mL treated cells suspension with 0.5 mL cell suspension without heat treatment, respectively. The resulting samples with a range of viable cell percentages were prepared for subsequent staining.

### 2.4. Flow Cytometric Analysis

Analysis was performed on a four-laser BD LSR II flow cytometer (BD Biosciences, San Jose, CA). The Hoechst 33342 dye was excited at 355 nm, and its emission fluorescence was detected using 440/30 nm (Hoechst 33342-Blue) and 670/40 (Hoechst 33342-Red) filter systems. PI was excited at 488 nm, and its fluorescence was detected using a 610/20 nm filter for dead cell exclusion. The flow data was analyzed using FlowJo Version 8 software (TreeStar, Ashland, CA, USA).

## 3. Results

**Figure 1:**
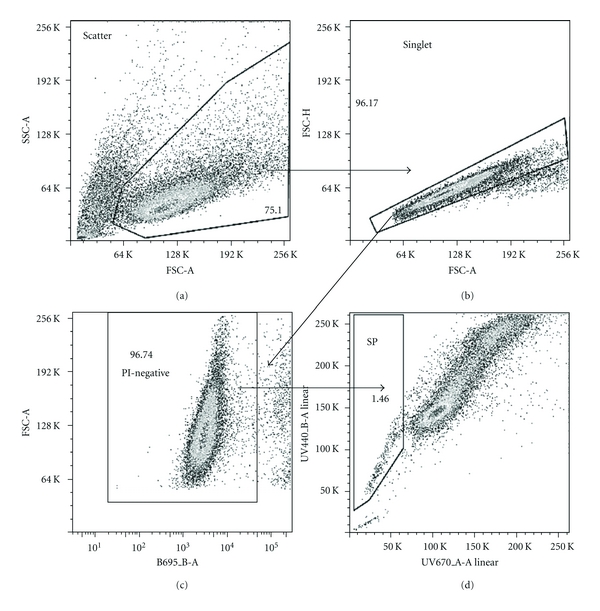
Flow cytometric analysis of RPMI-8226 stained with Hoechst 33342 and gating strategy for SP analysis. Sequential gating is shown from (a) to (d). (a) Light scatter flow cytometry profile for cells based on forward scatter (FSC-A) related to size and refractive index, and side scatter (SSC-A) related to granularity. (b) Singlet gating based on FSC-H versus FSC-A. (c) Dead cells excluded by PI binding. (d) SP profile.

This study is based on our previously published results showing a convincing FCM pattern of stained RPMI-8226 cells with Hoechst 33342, where a remarkable SP of about 20% presented with a clear gap existing between SP and non-SP cells [10]. Thus, we decided to harness the RPMI-8226 myeloma cell line to explore the factors affecting SP%. The gating strategy used for the present study is shown in Figure 1. The disappearance of SP cells observed in verapamil as well as FTC-treated samples was useful for the gating of SP in flow scattergrams.

### 3.1. Effect of Cell Culture Time Course

**Figure 2:**
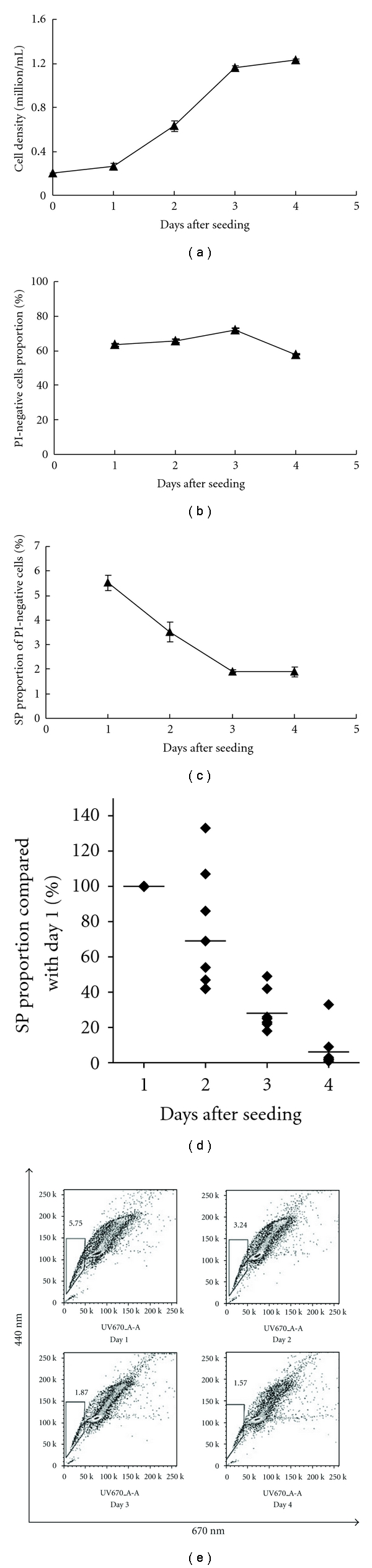
SP analysis of RPMI-8226 cells maintained in culture conditions for the indicated days. (a) Cell growth curve; (b) viable cell proportion determined by PI staining by flow cytometry; (c) SP proportion over the culture day; (d) SP% change with time after subculture; (e) SP flow cytometry profiles changed over the culture day. Data are mean ± SD value of triplicate samples in an independent experiment. Bar indicates standard deviation. One of the three independent experiments is shown as representative data.

Previous studies suggested that SP and non-SP cells were in different cell cycle phases [16]. We hypothesized that cell growth associated with different phases of the cell cycle would affect SP and non-SP distribution. To test for this hypothesis, we formally examined how cell culture time would affect SP%. The RPMI-8226 cells continued to grow before reaching the plateau stage on Day 4 after subculture as shown in Figures 2(a) and 2(b). Cells subcultured for different days up to four days were used to determine SP% using the standard staining protocol. Surprisingly, we found that SP% was highest at Day 1, which was 5.1% and 2.3% for batch 1 and 2, respectively, and then reduced to 2.0% and 0.1% for batch 1 and 2 at Day 4, respectively (Figures 2(c) and 2(d)).

### 3.2. Effect of Hoechst 33342 Dye Concentration

**Figure 3:**
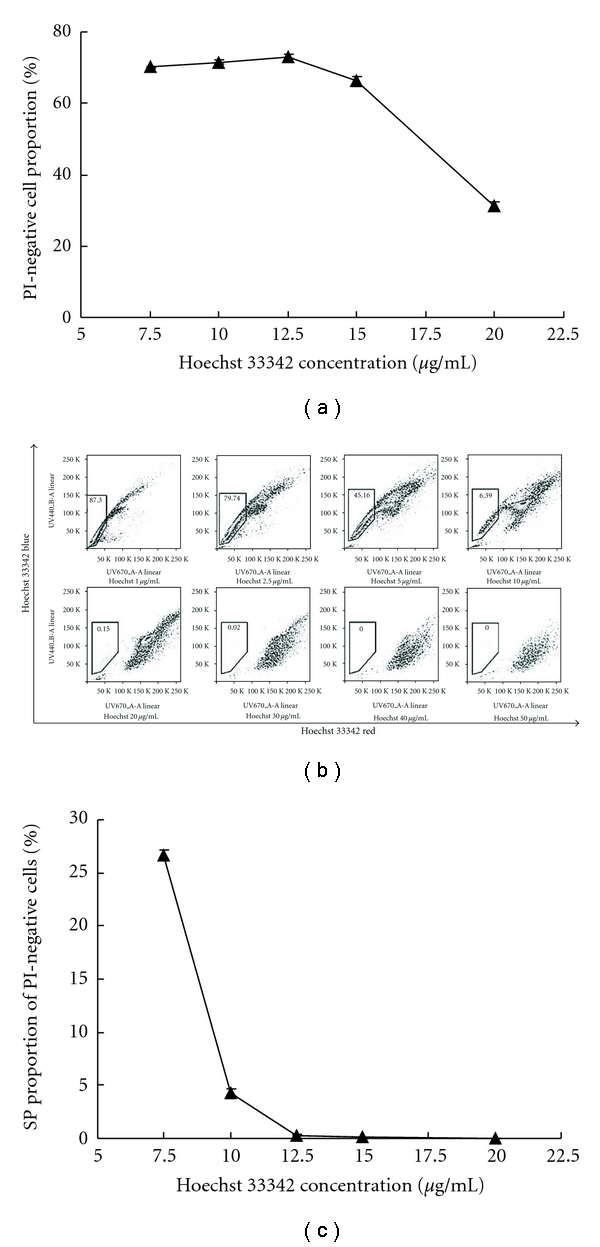
Flow cytometric analysis of RPMI-8226 cells stained with Hoechst 33342. (a) The proportion of viable cells started to decrease when dye concentration exceeded 15 *μ*g/mL. (b) Flow cytometry profiles of SP analysis changed over dye concentration from 1 to 50 *μ*g/mL. (c) In another experiment with Hoechst 33342 concentration ranging from 7.5 *μ*g/mL to 20 *μ*g/mL, SP% dropped sharply from 26.3% at 7.5 *μ*g/mL to 0.16% at 12.5 *μ*g/mL. Data are mean ± SD value of triplicate samples in an independent experiment. Bar indicates standard deviation. The experiment was repeated at least three times with similar results. One of the three independent experiments is shown as representative data.

We explored how the dye concentration influenced SP%. Cells were stained with a range of Hoechst 33342 concentration from 1 to 50 *μ*g/mL and then assessed for (i) cytotoxicity as measured by PI uptake indicating loss of plasma membrane integrity and (ii) change of SP%. RPMI-8226 cell viability was sensitive to Hoechst 33342 in a dose-dependent manner, which indicated that this DNA-binding dye was toxic to cells in higher concentrations (Figure 3(a)). The same gating method was applicable for samples with dye concentration <20 *μ*g/mL (Figure 3(b)) as there was only small change in flow cytometry profiles. However, we found that dye concentration had a pronounced effect on SP%. A sharp drop of SP% was observed when dye concentration increased from 7.5 *μ*g/mL (26.3%) to 10 *μ*g/mL (4.4%), which was followed by a much gentle reduction to 12.5 *μ*g/mL (0.16%) and onwards (Figure 3(c)). This significant decrease in SP% at 10 *μ*g/mL or higher concentrations was not due to the decrease in the percentage of live cells which were the same at around 70% (Figure 3(a)). Thus, we suggested that the optimum dye concentration for staining of RPMI-8226 cells to be 10 *μ*g/mL which was at the junction of the sharp and the gentle slope of SP% (Figure 3(c)).

### 3.3. Effect of Cell Viability

**Figure 4:**
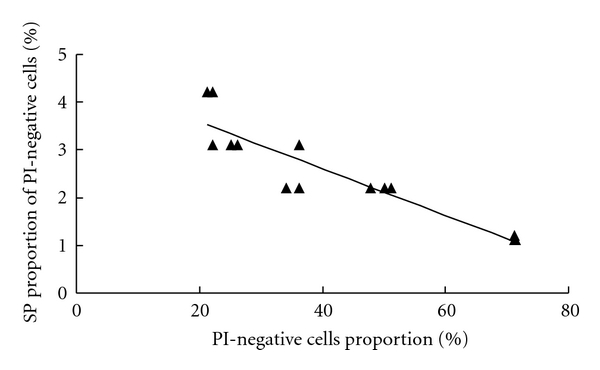
Relation between SP% and viable cell proportion in RPMI-8226. The experiment was repeated at least three times with similar results. One of the three independent experiments is shown as representative data.

By using a fixed number of live cells of the RPMI-8226, we continued to investigate the issue of whether the presence of a different number of dead cells in the staining medium would affect the staining of SP cells leading to changes of SP% when gated on live cell compartment. In this study, we manipulated the ratio of live/dead cells by mixing different numbers of heat-treated dead cells to a standard number of live cells and then measured the SP% of the live cells. The results shown in Figure 4 indicate that SP% was inversely proportional to the relative proportion of viable cell component. For instance, the mean value of SP% was 3.5% for samples with 20% live cells, in comparison to SP% of 1.1% when live cell proportion increased to 70%.

### 3.4. Effects of Staining Conditions

Next, we examined the effects of various components involved in the actual staining procedure including staining cell density, volume of staining solution, duration of staining incubation, interval between mixing during incubation, and the staining temperature. The results show that all of these factors contributed changes in SP%.

**Figure 5:**
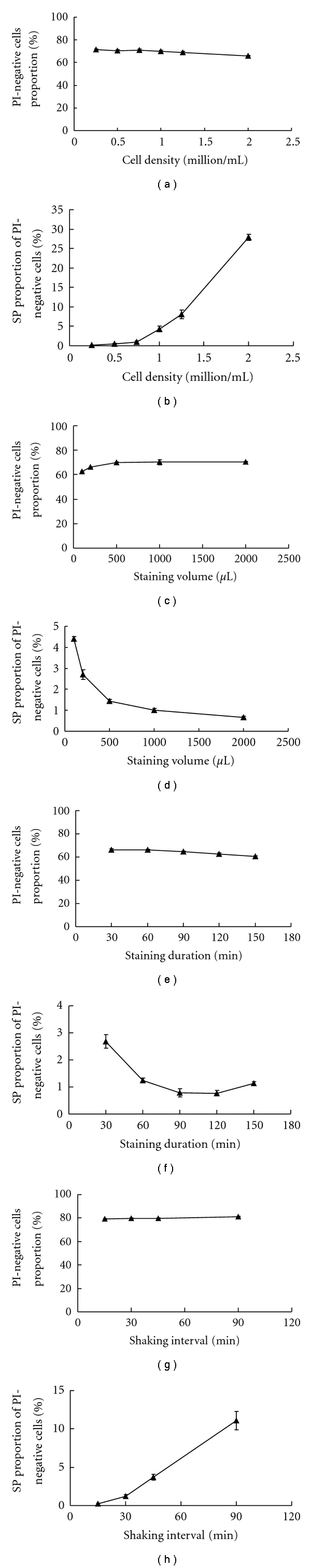
Effect of staining conditions on viable cell and SP%. (a) Live cell proportion remained relatively stable when staining cell density increased. Cell density ranged from 0.25 × 10^6^ to 2.00 × 10^6^ cells/mL. (b) SP percentage increased with increasing cell density. (c) Live cell proportion remained stable when staining volume increased from 100 to 2000 *μ*L. (d) SP percentage decreased with increasing staining volume. (e) The viable cell proportion prior to flow cytometry analysis was stable when increasing staining duration from 30 min to 150 min. (f) SP percentage decreased with increasing staining duration and plateaued after 90 min. (g) The viable cell proportion prior to flow cytometry analysis was stable when mixing interval time changed from 15 to 90 min. (h) SP percentage increased with the decrease of mixing interval. Data are mean ± SD value of triplicate samples in an independent experiment. Bar indicates standard deviation. The experiment was repeated at least three times with similar results. One of the three independent experiments is shown as representative data.

While examining staining cell density, the viability of stained cells was stable at around 70% for staining cell density from 0.5 × 10^6^ to 2 × 10^6^ cells/mL (Figure 5(a)). No SP cells were observed at the staining density of 0.75 × 10^6^ cells/mL or lower. The SP% increased sharply when the staining cell concentration increased to 1.5 × 10^6^ cells/mL or above (Figure 5(b)). 

Varying the duration of staining incubation showed that there was a constant SP% when the incubation was at least 90 min. Reducing the incubation period resulted in an increase of SP% while there was no change of viable cell proportions. Finally, we investigated the effects of the length of shaking intervals during the incubation using 90 min as the standard incubation period. As shown in Figure 5(h), no SPs were detected with frequent shaking of every 15 min or less, and then SP% proportionally increased in association with increasing shaking interval time. The result showed that a frequent mixing of the cells every 15 minutes may dramatically result in negative staining results. It is therefore important to follow a standard protocol of either a mixing interval of every 30 min or 45 min. 

In summary, in order to obtain a consistent SP%, it is important to observe carefully all the above-mentioned experiment requirements of viable cell proportion, dye concentration, staining cell density, incubation duration, staining volume, and mix interval, as well as the important factor of the time after subcultured.

## 4. Discussion

It has been postulated that cancer stem cells (CSCs) resist current drug therapies and repair DNA after radiation treatment more efficiently than their differentiated, daughter cells and that these CSCs are responsible for the recurrence of tumours after treatment [17–20]. Similarly we have previously published that these CSCs survive standard chemotherapeutics in multiple myeloma [10]. It is therefore likely that the only way to cure cancer is to target against the CSCs. 

It has been reported that the number of companies devoted to research into novel medicines targeting CSCs doubled from 2007 to 2008 [21]. In addition, patents covering developments in CSCs doubled to about 70 in 2007 [21]. Although there are only one or two drugs targeting CSC molecules currently in Phase II or Phase III/IV clinical trials, there are thirteen drugs in Phase I trials, with many more in the development pipeline. All these point to the fact that development of a robust drug screening platform that focuses on testing of effective compounds or medicines targeting CSCs is urgently required and could have very significant clinical impacts. 

Few prospective systematic studies have examined the factors influencing SP% in normal tissues or cancer cells. In this study, using the human myeloma cell line RPMI-8226 as a convenient cellular model system *in vitro*, we systematically explored multiple factors, namely, Hoechst 33342 dye concentration, cell density, staining duration, staining volume, mix interval during staining, and cell viability prior to enumerating SP cells by FCM analysis. We have highlighted the large variability in SP% which is obtained when different conditions are used and have optimised the technical aspects of staining SP cells. With regards to cell viability, a previous study has also shown that, for consistent SP staining, the cell count before the Hoechst staining should include all cells ranging from dying, dead, to live cells [14]. The same group also suggested that Hoechst concentration and toxicity curves should be independently determined for each new tissue or cell type being tested [14]. 

Importantly, we found that the time after cell subculture is an important factor affecting the SP% which has not been reported previously. While this factor will not affect the staining of SP in primary tissues, we envisage that a drug screening platform targeting SP for various cancer types will utilize stable cell lines. Thus, this new finding should lead to the modification of the preparation of cell samples for drug screening purposes. Furthermore, since RPMI-8226 harbors a rich and constant source of SP cells, it could be used as the positive control for this platform and for the detailed monitoring of the day-to-day variation of Hoechst staining performance.

## Abbreviations

SP: Side population
HSC: Hematopoietic stem cells
FACS: Fluorecence activated cell sorting
FCM: Flow cytometry
FTC: Fumitremorgin C
FBS: Fetal bovine serum
HBSS: Hanks' Balanced Saline Solution
PI: Propidium iodide.
